# Efficacy and safety of Buyang Huanwu decoction in the treatment of varicose veins of the lower extremities: A protocol of randomized controlled trial

**DOI:** 10.1097/MD.0000000000024663

**Published:** 2021-02-26

**Authors:** Chuanyong Li, Weijian Fan, Zhichang Pan, Guangfeng Zheng, Qiang Zhang, Jianjie Rong

**Affiliations:** Department of Vascular Surgery, Suzhou TCM Hospital Affiliated to Nanjing University of Chinese Medicine, Suzhou, P R China.

**Keywords:** Buyang Huanwu decoction, protocol, randomized controlled study, varicose veins

## Abstract

**Background:**

Varicose veins of the lower extremities are common chronic venous diseases in the clinic. Although Western medicine has various surgical methods to treat varicose veins in the lower extremities, there are still a variety of complications. Some studies have shown that Buyang Huanwu decoction treatment of varicose veins of the lower extremities has a certain effect, and can reduce the occurrence of postoperative complications, but there is no evidence of evidence-based medicine. The research carried out in this scheme is to systematically evaluate the efficacy and safety of Buyang Huanwu decoction in the treatment of varicose veins in the lower extremities, and to provide reliable evidence for guiding clinical practice.

**Methods:**

This is a randomized, double-blind, placebo-controlled, parallel-group clinical trial, which studies the effectiveness and safety of Buyang Huanwu decoction in the treatment of varicose veins of the lower extremities. The patients are randomly and evenly divided into treatment group and control group, the former one is given Buyang Huanwu decoction and the latter one is given placebo. The study will last 49 days, including a 7-day washout period, 14-day intervention and 28-day follow-up, focusing on its efficacy and safety indicators. Observation indicators include: TCM syndrome score, Venous Clinical Severity Score (VCSS), Venous Disability Scote (VDS), Aberdeen Varicose Vein Questionnaire (AVVQ), Hemorheology Indicators, Adverse Reactions, etc. Data analysis is performed using SPSS 25.0 software.

**Discussion:**

This study will evaluate the effectiveness and safety of Buyang Huanwu decoction and provide clinical evidence for the treatment of varicose veins of the lower extremities.

**Trial registration:**

OSF Registration number: DOI 10.17605/OSF.IO/WGJXT.

## 1 Introduction

Varicose veins (VVs) of the lower extremities are common chronic peripheral vascular diseases with incidences of 20% to 30%. The incidence of varicose veins in females is higher than that in males. The most common lesion was the great saphenous vein.^[1]^ The disease has a long course and can easily cause many complications such as chronic ulcer, thrombophlebitis, and vein rupture.^[2]^ Patients may have lower limb edema, pigmentation, deformity, bleeding, and other symptoms, which seriously affect the quality of their lives.^[3]^ Studies have shown that people who are engaged in manual work, especially standing work, are more likely to have varicose veins of the lower limbs.^[4]^ To date great progress has been made in the study of the pathogenesis of varicose veins of the lower extremities, which is closely related to vascular smooth muscle cells (VSMCs). Apoptosis, proliferation, and vascular remodeling of vascular smooth muscle cells can affect the veins of the lower extremities.^[5–7]^ For a long time, the traditional surgical treatment is high ligation of the junction of the great saphenous femur and exfoliation of the great saphenous vein, which is considered to be the gold standard of treatment.^[8]^ Although there are various surgical methods for the treatment of varicose veins of the lower extremities, they cannot effectively avoid postoperative limb swelling, subcutaneous hematoma, ecchymosis, saphenous nerve injury, and other complications, which brings inconvenience to the postoperative recovery of patients.^[9,10]^ Various postoperative complications not only affect limb movement, leading to delayed wound healing, but also cause psychological burden to patients, so it is particularly important to find other methods to treat or prevent the occurrence of postoperative complications.

Varicose veins of the lower extremities belong to the category of “muscle tumor” in Chinese medicine, and the pathogenesis is blood stasis as the core. In recent years, clinical studies have found that Chinese medicine plays an important role in the treatment of varicose veins of the lower extremities and the prevention of postoperative complications, which is conducive to the recovery of the disease and shortens the length of hospitalization.^[11]^ Buyang Huanwu decoction comes from *Correction on Errors in Medical Classics*. Its prescriptions are composed of Radix Astragali seu Hedysari, Lumbricus, Flos Carthami, Radix Paeoniae Rubra, Radix Angelicae Sinensis, Semen Persicae, and Rhizoma Ligustici Chuanxiong, aiming at invigorating vital energy and promoting blood circulation for removing obstruction in collaterals. Buyang Huanwu decoction can regulate lipid metabolism, stabilize plaque, and has a good effect on preventing atherosclerosis.^[12]^ With acupuncture and moxibustion treatment, it can well improve the clinical symptoms of patients with stroke sequelae.^[13]^ At the same time, Buyang Huanwu decoction has little clinical adverse effects. Therefore, we plan to evaluate the effectiveness and safety of Buyang Huanwu decoction through this randomized controlled trial, and provide a treatment plan for clinical treatment of varicose veins of the lower extremities.

## 2 Materials and methods

### 2.1 Study design

**Figure 1:**
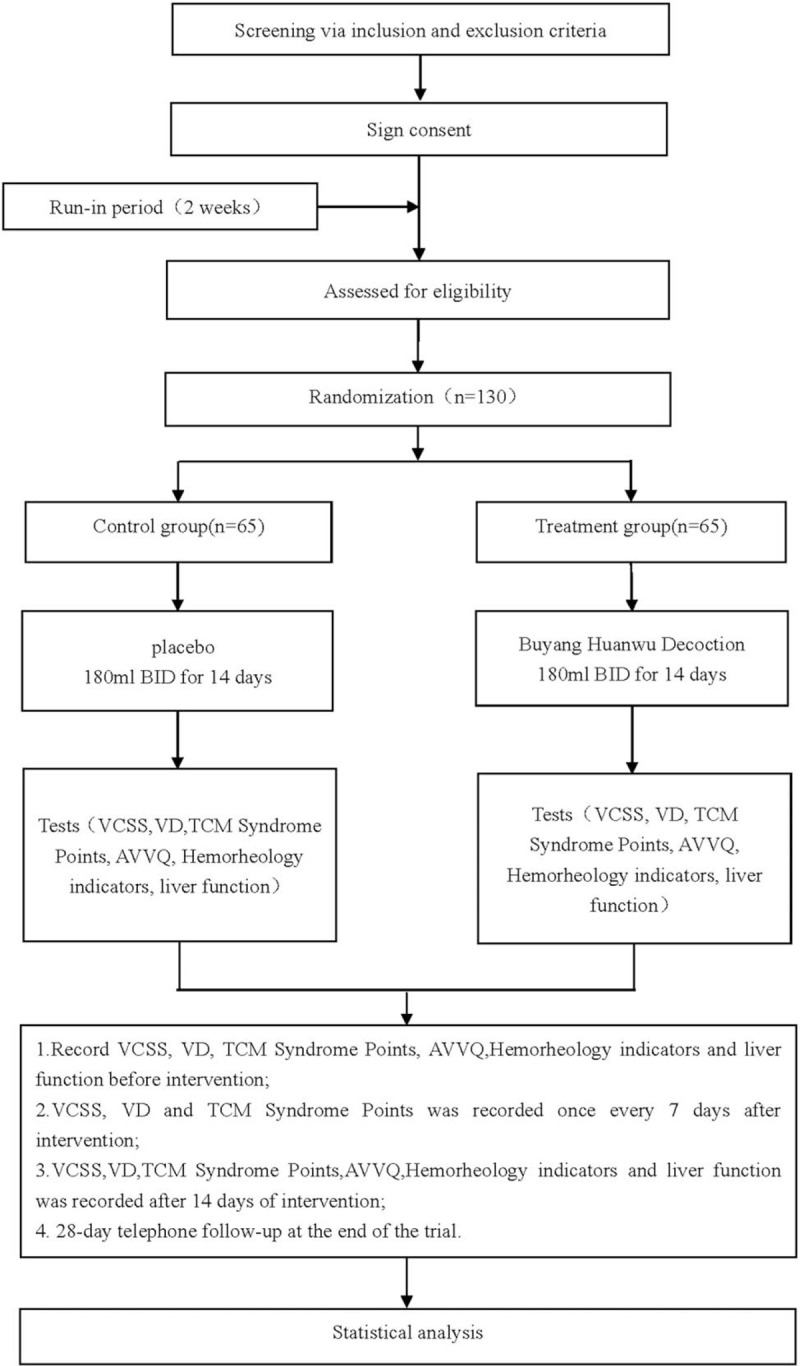
Flow diagram.

This is a randomized, double-blind, placebo-controlled, parallel-group clinical trial that indicates to study the effectiveness and safety of Buyang Huanwu decoction in the treatment of varicose veins of the lower extremities. The whole experiment will follow the comprehensive test report standard. And the flowchart is shown in Figure 1.

### 2.2 Ethics and registration

This research protocol complies with the Declaration of Helsinki. Furthermore, it is approved by the clinical research ethics committee of our hospital and registered on OSF (registration number: DOI 10.17605/OSF.IO/WGJXT). Before randomization, all patients need to sign a written informed consent and they are free to choose whether to continue the trial at any time.

### 2.3 Patients

Inclusion criteria: ①Meet the diagnostic criteria of western medicine for varicose veins of the lower extremities;^[14]^②People age between 20 and 70; ③All patients signed informed consent, and the research materials are complete.

Exclusion criteria: ①Those with insufficiency of important organs such as liver, kidney, heart and lung; ②Those suffer from other serious diseases such as malignant tumors; ③Those with severe mental illness; ④Those with deep vein thrombosis; ⑤Patients with allergies or contraindications to the medication used in this study; ⑥Patients who are unable to understand the research plan after explanation or unwilling to participate.

Exit criteria: ①Those who have serious adverse events or serious complications are not suitable for the next test; ②Those who have poor compliance and affect the judgment of effectiveness and safety; ③For any reason, the subject asked to withdraw from the trial.

### 2.4 Sample Size Calculation

This study is a pilot clinical trial. Based on the analysis and calculation of the relevant literature published in the past 10 years, the efficiency of western medicine is 75.47%, and the effective rate of Chinese medicine combined with western medicine is 96.23%. The sample content estimation formula is used for comparison of multiple sample rates. Set the significance test level α = 0.05, then μ_α_ = 1.96, 1-β = 0.9, μ_β_ = 1.28, δ = π1-π2. The sample content estimation formula for comparing 2 sample rates: $N_{1}=N_{2}={\left(\frac{\mu_{\alpha }+\mu_{\beta }}{\delta }\right)}^{2}[\pi_{1}(1-\pi_{1})+\pi_{2}(1-\pi_{2})]$

The calculated sample size of each group is 54 cases. Considering factors such as shedding, it is expanded by about 20%, and 130 patients need to be included. According to the order of visits, the random number method was used to divide the patients into a control group and an observation group, with 65 cases in each group.

### 2.5 Study design

This study will recruit patients who come to the outpatient clinic to screen and happen to meet the criteria. The patients and their families will approve the study plan and sign an informed consent form. The patients are randomly assigned to the treatment group and the control group. Among them, the treatment group is given Buyang Huanwu decoction (Radix Astragali seu Hedysari 30 g, Lumbricus 6 g, Flos Carthami 6 g, Radix Paeoniae Rubra 10 g, Radix Angelicae Sinensis 10 g, Semen Persicae 6 g, and Rhizoma Ligustici Chuanxiong 6 g), 1 bag at a time (180 ml), twice a day; The control group is given a placebo. The packaging, color, taste, dosage form and size of the placebo are exactly the same as the treatment group. The 2 groups of patients will receive the same routine care and basic treatment, control the patient's blood sugar, blood pressure, blood lipids, etc., and receive health education. If necessary, the attending doctor can adjust the plan according to the patient's condition. All intervention methods will be recorded in detail. Used for final result analysis.^[15]^ We will set up a dedicated drug administrator, and the nurse will be responsible for preparing drugs. Therefore, this study is not blind to nurses, but to researchers, patients and statisticians. All patients will receive 14 days of treatment. Before and after treatment and on the 7th day of treatment, the health status of each patient will be assessed, including efficacy indicators and safety indicators, and all patients will be followed up for 28 days by telephone. Follow-up content includes cardiovascular events and rehospitalization.

### 2.6 Evaluation criteria and judgment of curative effect

1. Main observation indicators: Venous Clinical Severity Score; Venous Dysfunction Score^[16]^; TCM Syndrome Points;
2. Secondary observation indicators: Aberdeen Varicose Vein Questionnaire; Hemorheology indicators (including whole blood viscosity, plasma viscosity, hematocrit, fibrinogen and erythrocyte sedimentation rate (ESR));
3. Adverse reactions: Including abnormal liver function and any uncomfortable symptoms (such as dizziness, nausea, etc.) during treatment.

### 2.7 Data collection and management

Before the start of treatment, 7 days after the start of the treatment, and at the end of the treatment, collect data according to the evaluation criteria. 28 days after the end of the treatment, each patient will be followed up by outpatient or telephone. The detailed record of the follow-up information cannot be collected. Two assistants cooperate to collect. Personal information about potential participants and registered participants will be collected, shared and stored in an independent storage room to protect confidentiality before, during and after the test. Access to the database is limited to researchers in this research group.

### 2.8 Statistical analysis

In this study, SPSS25.0 statistical analysis software is used for data analysis, and the measurement data is represented by ($\bar{x}\pm s$); For data conforming to normality and homogeneity of variance, the group *t* test is used between groups, and the paired *t* test is used within the group; the rank sum test is used for those that does not conform to the normal distribution; the Chi-Squared test is used for count data. *P* < .05 is statistically significant.

## 3 Discussion

The clinical manifestations of varicose veins of the lower extremities are chronic venous insufficiency (CVI), the main symptoms are varicose veins, edema, skin pigmentation and ulcers, etc.,^[17]^ it is a common type of disease in vascular surgery,^[18]^ more common in all parts of the world.^[19]^ Varicose veins of the lower extremities often occur in workers or standing for a long time, and its risk factors include pregnancy, smoking, and overweight.^[20]^ The veins of the lower extremities are equipped with effective tube walls, contracted vascular smooth muscles and qualified valves to withstand pressure and allow deoxygenated blood to flow to the heart in 1 direction.^[21]^ When some factors cause it to exceed the body's own regulation, it will manifest as symptoms such as varicose veins in the lower limbs. If effective methods are not taken, it will lead to the occurrence of ulcers in the lower limbs, which not only affects the health of the patient, but also greatly reduces their quality of lives.^[22]^ Surgery is the most common method for the treatment of varicose veins of the lower extremities. The clinical effect is definite,^[23]^ but patients are prone to various complications after operation, such as lower extremity phlebitis, lower extremity venous thrombosis, etc.,^[24,25]^ which seriously affect the quality of life of patients, and even threaten life safety, so it is of great significance to find other suitable treatment methods.

Traditional Chinese medicine treatment of varicose veins of the lower extremities is mainly based on blood circulation and stasis removal drugs.^[26]^ But Buyang Huanwu decoction has the effect of replenishing Qi and activating blood. Modern pharmacological studies have found that Buyang Huanwu decoction has the effects of inducing angiogenesis, inhibiting cell apoptosis, and protecting nerves.^[27–29]^ It can significantly increase the levels of element binding protein and vascular endothelial growth factor, and significantly reduce inflammation,^[30,31]^ at the same time has the effect of anti-atherosclerosis,^[32,33]^ it can be seen that Buyang Huanwu decoction can play its unique advantages in treating patients with varicose veins of the lower extremities, and has fewer adverse reactions.

This randomized controlled trial is to verify that Buyang Huanwu decoction can significantly improve the clinical symptoms of patients with varicose veins and improve vascular function. Since there is no high-quality randomized controlled trial to evaluate the efficacy and safety of Buyang Huanwu decoction for patients with varicose veins of the lower limbs, it is very meaningful for us to carry out this study.

## Author contributions

**Data collection**: Chuanyong Li, Weijian Fan.

**Data curation:** Chuanyong Li, Weijian Fan.

**Funding acquisition:** Jianjie Rong.

**Funding support**: Jianjie Rong.

**Resources:** Weijian Fan, Zhichang Pan.

**Software:** Qiang Zhang, Jianjie Rong.

**Supervision:** Zhichang Pan, Guangfeng Zheng.

**Writing – original draft:** Chuanyong Li, Weijian Fan.

**Writing – review & editing:** Chuanyong Li, Jianjie Rong.
